# Quality of food-frequency questionnaire validation studies in the dietary assessment of children aged 12 to 36 months: a systematic literature review

**DOI:** 10.1017/jns.2017.12

**Published:** 2017-05-08

**Authors:** Amy Lovell, Rhodi Bulloch, Clare R. Wall, Cameron C. Grant

**Affiliations:** 1 Discipline of Nutrition, Faculty of Medical and Health Sciences, University of Auckland, Auckland, New Zealand; 2 Discipline of Nutrition, Faculty of Medical and Health Sciences, University of Auckland, Auckland, New Zealand; 3 Discipline of Nutrition, Faculty of Medical and Health Sciences, University of Auckland, Auckland, New Zealand; 4 Department of Paediatrics: Child and Youth Health, University of Auckland, Auckland, New Zealand; 5 Centre for Longitudinal Research He Ara ki Mua, University of Auckland, Auckland, New Zealand; 6 Starship Children's Hospital, Auckland District Health Board, Auckland, New Zealand

**Keywords:** Food-frequency questionnaires, Infants, Validity, Dietary assessment methods, 24-HR, 24-h recall, EURRECA, EURopean Micronutrient RECommendations Aligned, WFR, weighed food record

## Abstract

A child's diet is an important determinant of growth and development. Because of this, the accurate assessment of dietary intake in young children remains a challenge. A systematic search of studies validating FFQ methodologies in children 12 to 36 months of age was completed. English-language articles published until March 2016 were searched using three electronic databases (MEDLINE, EMBASE and CINAHL). Quality assessment of the identified studies was carried out using The Reduced Summary Score and EURopean micronutrient RECommendations Aligned (EURRECA) scoring system. Seventeen studies were included and categorised according to whether they reflected long-term (≥7 d) or short-term (<7 d) intake, or used a biomarker. A total score for each micronutrient was calculated from the mean of the correlation coefficients weighted by the study quality score. At least three validation studies per micronutrient were required for inclusion. Fifteen studies (83 %) that considered validity of the FFQ in assessing nutrient intakes had quality scores from 2·5 to 6·0. Of those, ten (67 %) studies found FFQ to have good correlations in assessing dietary intake (>0·4). Of the nutrients with three or more studies available, FFQ validated using a reference method reflecting short-term intake had a good weighted correlation for Ca (0·51), and acceptable weighted correlations for vitamin C (0·31) and Fe (0·33). Semi-quantitative FFQ were shown to be valid and reproducible when estimating dietary intakes at a group level, and are an acceptable instruments for estimating intakes of Ca, vitamin C and Fe in children 12 to 36 months of age.

The accurate description and measurement of dietary intake is a necessary step in determining the nutritional adequacy of diets in individuals or a population^(^^1^^)^. Having valid and reliable assessment tools is essential to increase our understanding of the relationship between dietary intake and health outcomes, and our understanding of the dietary determinants of nutritional status^(^^2^^)^.

Food and nutrient intakes are estimated via dietary assessment methods that differ according to a study's aims and objectives, skills of the study population, accuracy of the required dietary data, study resources and study design^(^^3^^)^. Most epidemiological studies use variations of the FFQ, which can be validated using biomarkers or tools that measure daily dietary intake^(^^5^^)^. The FFQ has an advantage of being an inexpensive method of obtaining data from a large number of participants, with a relatively low respondent burden and can be used to estimate an individual's average consumption over an extended period of time^(^^3^^,^^6^^)^.

There is no definitive ‘gold standard’ in dietary assessment, nor is there a ‘gold standard’ for assessing the validity of FFQ^(^^7^^)^. Therefore estimation of a tool's relative validity relies upon a comparison with a superior and preferably independent technique, known as comparative validation^(^^3^^)^. Here, weighed food records (WFR) and 24-h recalls (24-HR) are commonly used due to their greater precision in the quantification of intake^(^^3^^)^. Factors that may affect the validity of a diet questionnaire have been reviewed^(^^5^^,^^8^^)^.

Early childhood is a life phase where the assessment of dietary intake is particularly challenging. Measurement of energy and nutrient intakes in young children is affected by unique respondent and observer considerations, making the collection of accurate and reliable dietary intakes difficult^(^^1^^)^. Young children aged 12 to 36 months, have highly variable diets that are characterised by rapidly changing food habits and transitions in dietary patterns, and often not all food served to an infant is consumed in its entirety^(^^9^^–^^12^^)^. The acquisition of dietary intake information for children less than 7 years of age is dependent upon surrogate reporters, e.g. parents, caregivers and external caretakers^(^^1^^,^^13^^)^. Therefore, the accuracy of dietary assessment in this age group depends on an adult's ability to reliably report on their intake, with previous evidence suggesting that parents can provide a more reliable report on foods consumed in the home setting, rather than away from home^(^^1^^,^^13^^)^.

As a consequence of these methodological challenges, the number and type of validated tools available to assess the dietary intake of young children, particularly children 12 to 36 months of age, are limited. The aim of this systematic literature review was to describe and assess the quality of studies reporting on the validity of FFQ as a method for assessing food and nutrient intakes or dietary patterns in 12- to 36-month-old children.

## Methods

### Protocol registration

The inclusion and exclusion criteria, and analysis methods were specified in advance in a documented protocol. This protocol was not registered with PROSPERO^(^^14^^)^ as it is an assessment of the quality of validation studies and does not report on a health-related outcome.

### Eligibility criteria

**Fig. 1.**
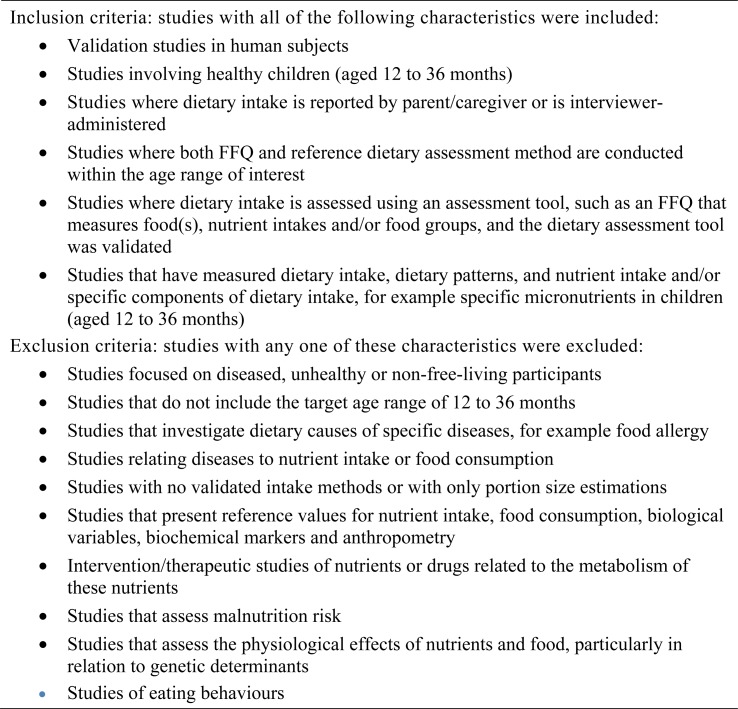
Inclusion and exclusion criteria used to select studies for inclusion in the systematic review.

Studies that evaluated the validity of FFQ in the assessment of dietary intake, food(s), and dietary patterns with a reference dietary assessment tool (e.g. 24-HR, diet records, diet histories, WFR and biomarkers) in healthy children aged 12 to 36 months and met all the inclusion criteria (Fig. 1.) were included in the review. Randomised controlled trials were not available; therefore analytical study designs were limited to prospective and retrospective cohort studies. Case series, case reports and case–control studies were excluded due to the high potential for bias.

### Information sources

Studies were identified via searching online databases, hand-searching reference lists of original articles, and cited reference searches. The search focused on relevant studies published before March 2016 and was limited to those published in English, without limits on time frame or country. Grey literature was also considered.

### Search strategy

A literature search was applied to MEDLINE (1946 to present), EMBASE (1980 to present) and CINAHL (1937 to present) electronic databases, and Google Scholar. Medical Subject Headings (MeSH), MeSH major topics, and free text terms were developed under four group headings in MEDLINE and EMBASE databases. The MeSH search terms used in the search were developed under four group headings: (1) infant (12–36 months), e.g. toddler, preschool*, child, infant, newborn*, pre-school*, babies, baby, kindergarten, children under 2, children under 3; (2) diet, e.g. nutrition, dietary pattern, food intake, diet quality, infant nutrition, child nutrition, nutritional assessment, eating pattern, nutritional status, feeding behaviour, food combination, childhood diet, infant food; (3) dietary assessment, e.g. diet surveys, questionnaires, instrument, dietary intake methods, assess*, evaluat*, dietary intake methods, nutrition surveys; (4) dietary assessment tool, e.g. food frequency questionnaire, FFQ; (5) instrument validation, e.g. validity, reproducibility, correlation coefficient, reliability, validation studies, replication stud*, correlation stud*, repeatability. Key words and combinations were identified in free text, article titles and abstracts, and were used to perform a comprehensive search of the databases. Search terms and strategies were adapted for use in other databases and were peer reviewed. All retrieved articles were sent to Refworks^®^ (version 4.4.1237; ProQuest LLC) where duplicates were removed.

### Study selection

Two reviewers (A. L. and R. B.) determined a study's eligibility in an independent, unblinded and standardised manner. Systematic literature reviews were not included in the analysis. Titles and abstracts were reviewed to assess whether they met the inclusion criteria for full-text review (Fig. 1). Disagreements between reviewers were resolved by consensus, or if the decision on study inclusion or exclusion were unclear, the full text was obtained. In studies where the age range of participants was included, but was much wider than 12 to 36 months, e.g. 2 to 9 years, the reviewers attempted to obtain results from authors specific to the age range of interest. Full-text articles that fulfilled all criteria for inclusion were reviewed in a second screening process as the definitive step for inclusion.

### Data collection process

A data extraction sheet based on examples found in the selected literature was developed. One review author (A. L.) extracted key data into a prepared table, which was checked by a co-author (R. B.). Any disagreements were resolved through discussion between the review authors (A. L. and R. B.), and if no agreement could be reached a prearranged third reviewer was asked to arbitrate (C. W.). Direct contact via email was made with four authors to obtain information in addition to that which could be abstracted from the published paper. In all four cases this request was for information within the age range of interest (12 to 36 months) from a study that reported data over a wider age range. One follow-up email was sent if no response was received. No authors responded with data from their studies specific to the age range of interest.

### Data items

A concise overview of the seventeen included studies is shown in Table 1^(^^11^^,^^15^^–^^24^^,^^26^^–^^31^^)^. The areas of interest included: population characteristics (size, age, location, ethnicity), FFQ characteristics (food groups, food items, consumption interval, administration method, portion estimation, number of FFQ administered, and FFQ re-test interval), reference method used, outcome measures (validity, reproducibility) and the statistics employed to assess validity between two methods or reproducibility of the FFQ.

**Table 1.** Characteristics of included studies evaluating long-term or short-term nutrient intake, or biomarker, food or food group

Reference (year), country	Population number (girls/boys), age range	FFQ food groups	FFQ food items	Consumption interval	FFQ type	Administration method	Portion estimation	No. of FFQ administered	FFQ interval (retest)	Reference method (characteristics)	Statistics used for validation	Correlation coefficient
Long-term intake												
Andersen *et al.*^(^^11^^)^ (2003), Norway	64 (26/37), 12 months	15	140	Previous 14 d	Semi-quantitative	Self-administered (parent)	Household units Photographs: 16 photographs (small, medium, large)	1	NA	7 d WFR 1–2 weeks following FFQ 1-week interval between FFQ and WFR Two periods: 4 d (rec. 1), 3 d (rec. 2)	Spearman CC, energy-adjusted CC, cross-classification, Bland–Altman	Absolute intake: 0·47*, 0·50† Nutrient density: 0·51*, 0·50† Food intake: 0·62†
Andersen *et al.*^(^^15^^)^ (2004), Norway	187 (88/99), 24 months	15	125	Previous 14 d	Semi-quantitative	Self-administered (parent)	Household units Photographs: 16 photographs (small, medium, large)	1	NA	7 d WFR 1–2 weeks following FFQ	Spearman's CC, energy-adjusted CC, Bland–Altman, cross-classification	Absolute intake: 0·36*, 0·38† Nutrient density: 0·53*, 0·52† Food intake: 0·48†
Short-term intake												
Iannotti *et al.*^(^^16^^)^ (1994), USA	17 (8/9), 2–4 years	NR	111	Previous 7 d	Willet (semi-quantitative)	Interviewer-administered	Free text for portion sizes	1	NA	3 d WFR Parent-completed Food and drink not consumed Recorded data checked	Pearson's CC	0·37 *
Blum *et al.*^(^^17^^)^ (1999), USA	233, 1–5 years	NR	84	4 weeks	Adapted Willet (semi-quantitative)	Self-administered (parent)	Age-appropriate	2	1 month	Three 24 h recalls Over 4 weeks (2 weekdays, 1 weekend) Month between HFFQ1 and HFFQ2 Interviewer-administered	Pearson's CC, adjusted energy intake,	0·52 (all) 0·51 (1–2 years)
Parrish *et al.*^(^^18^^)^ (2003), Canada	68, 1–3 years	NR	111	Previous 12 months	Willett (semi-quantitative)	Self-administered (parent)	NR	2	NA	24HR (x3 or 4) Completed prior to FFQ 1 × 24HR every 3 months Interviewer-administered Biomarkers (*n* 38) Total lipids calculated (vitamins C, D and E, retinol, β-carotene)	Energy-adjusted, Pearson CC, Spearman's CC	0·33*: 1 year 0·30*: 2 years 0·27*: 3 years 0·32*: all years
Marshall *et al.*^(^^19^^)^ (2003), USA	240 (119/121), 6 months–5 years	7	NR	Previous week	Quantified beverage frequency questionnaire	Self-administered (parent)	Beverage consumption in servings per week	18	NA	3 d food and beverage diary 1 weekend + 2 weekdays Parent-completed	Spearman's CC, weighted κ	Ca 0·62* all 0·64* drinkers Vitamin D 0·63* all 0·70* drinkers
Williams & Innis^(^^20^^)^ (2005), Canada	148, 8–26 months	21	191	Previous 2 weeks	Quantitative	Interviewer-administered	NR	1	NA	3 d FR (24HR completed prior) Parent-completed 2 weekdays, 1 weekend Biomarkers Serum markers of Fe Food pictures, 3D models, household measures	Spearman's CC, energy-adjusted CC	0·63* 0·71* (energy adjusted)
Marriott *et al.*^(^^21^^)^ (2009), UK	50 (23/27), 12 months	NR	78	Previous 28 d	Semi-quantitative	Interviewer-administered	Household measures, food models	1	NA	4 d WFR following FFQ	Spearman CC, energy-adjusted CC, Bland–Altman, LOA	0·49* 0·48* (energy adjusted)
Vereecken *et al.*^(^^22^^)^ (2010), Belgium	216 (112/104), 3·5 years (mean age)	77	NR	Previous 3 months	Quantitative	Self-administered (parent)	4–8 portion sizes for each item Open category	1	NA	Online dietary A_x_ tool Parent-completed online 3 non-consecutive days (1 weekend, 2 weekdays) over 2-week period	Spearman's CC, Bland–Altman	0·42*
Rankin *et al.*^(^^23^^)^ (2011), USA	753 (388/365), 6–60 months	NR	16 (6–20 months), 11 (20–60 months)	Previous 7 d	Semi-quantitative	Self-administered (parent)	NR	1	NA	3 d FD Over 72 h (1 weekend, 2 weekdays) After the FFQ	Spearman's CC	0·73*
D'Ambrosio *et al.*^(^^24^^)^ (2012), Canada	22, 12–35 months	10	97	Previous 4 weeks	HFFQ (semi-quantitative)	Interviewer-administered	Household measures, food models	2	1 month (*n* 14)	24HR Parent-completed over 2 weeks Completed 2 and 4 weeks following FFQ (same day as FFQ) Reliability in subset: 4 weeks following FFQ	Pearson's CC, Bland–Altman	0·41* (CC)
Watson *et al.*^(^^26^^)^ (2015), New Zealand	160, 12–24 months	16	91	Previous 4 weeks	Southampton Women's Survey FFQ (Quantitative)	Interviewer-administered	‘Palm’ sizes	2	4 weeks	5 d WFR Non-consecutive days randomly assigned Completed over 5 weeks	Pearson's CC, cross-classification, ICC, Bland–Altman	0·50 (unadjusted) 0·52 (adjusted)
Sochacka-Tatara & Pac^(^^27^^)^ (2013), Poland	143 (68/75)	95	95	Previous 4 weeks	Semi-quantitative	Self-administered (parent)	Household units	1	NA	24HR (x3) 3 consecutive days (average taken) Weekdays + weekends Interval between FFQ and 24HR ≤1 month Parent-completed	Spearman's CC, Bland–Altman	0·46*
Biomarker												
Orton *et al.*^(^^28^^)^ (2008), USA	404 (107/209), 1–11 years	NR	111	Previous year	Semi-quantitative	Self-administered (parent)	Commonly used portion sizes, e.g. slice of bread	1	NA	Biomarker Fatty acid composition measured in erythrocytes from blood sample	CC, linear model	0·10* (energy adjusted) 0·08* (% total fat from FFQ)
Food or food group intake												
Klohe *et al.*^(^^29^^)^ (2005), USA	77, 1–3 years	91	191	Previous month	Semi-quantitative	Self-administered (parent)	Serving sizes: small, medium, large, extra-large (as multiples of a medium serving size)	2	2 weeks	3 d DR (24HR + 2 d DR) 24HR: interviewer-administered Household measures, food models 2 d DR: weekend + week day	Spearman's CC, cross-classification	0·41*
Bel-Serrat *et al.*^(^^30^^)^ (2011), Europe	2508 (1244/1264), 2–9 years	43	NR	Previous month	Children's Eating Habits Questionnaire (non-quantitative)	Self-administered (parent)	NR	1	NA	24HR (x2) Computerised Non-consecutive days Interviewer-administered	Pearson's CC (de-attenuated), Spearman's CC, cross-classification, Kruskal–Wallis, Bland–Altman, LOA, weighted κ	0·25* (age 2–6 years)
Mills *et al.*^(^^31^^)^ (2015), New Zealand	153 (51/78), 12–24 months	16	91	Previous 4 weeks	Adapted Southampton Women's Survey FFQ (quantitative)	Interviewer-administered	‘Palm’ sizes	2	4 weeks	5 d WFR Non-consecutive days randomly assigned Completed over 5 weeks	Pearson's CC, cross-classification, ICC	0·61* 0·58†

3D, three-dimensional; 24HR, 24 h recall; CC, correlation coefficient; DR, diet record; FD, food diary; FR, food record; HFFQ, Harvard Service Food Frequency Questionnaire; ICC, intra-class correlation; LOA, limits of agreement; NA, not applicable; NR, not reported; rec., record; WFR, weighed food record.

* Mean.

† Median.

### Synthesis of results

Studies were classified into three categories based on the reference method applied to the validation study. This method has been previously reported and consisted of:

1. Long-term intake – the reference method covered ≥7 d.
2. Short-term intake – the reference method covered <7 d.
3. Biomarker – the reference method was a biomarker.

### Quality assessment

Following classification, the two reviewers (A. L. and R. B.) independently completed quality assessment of the included validation studies using the reduced summary score by Dennis *et al*.^(^^32^^)^ which assessed the quality of the nutrition information from the FFQ, and an additional scoring system developed by the EURopean Micronutrient RECommendations Aligned (EURRECA) network used in studies assessing nutrient intakes with the aim of including, excluding and weighting studies^(^^5^^,^^12^^)^. These scoring tools evaluated methodological quality of the identified studies and determined the extent to which a study addressed the possibility of bias in their design, conduct and analysis. This dual scoring system approach was used in a previous review of FFQ for assessing dietary intake in adolescents^(^^33^^)^.

Because of the heterogeneity between the dietary assessment methods used as the reference, study designs, populations, and duration of the study, only a narrative review of the literature was performed. A meta-analysis could not be conducted due to a lack of randomised controlled trials.

The summary score by Dennis *et al*.^(^^32^^)^ scores studies based on objective measures of quality dietary assessment. The reduced summary score with a maximum score of 8 was utilised for simplified quality assessment of the FFQ as seen in Tabacchi *et al*.^(^^33^^)^ Validation studies that had a reduced summary score of ≥5 were classified as being ‘high quality’ and scores <5 as ‘low quality’. This scoring tool was used for all included studies. The EURRECA^(^^5^^)^ scoring system was only applied to studies that assessed nutrient intakes. Summary scores range from 0 (poorest quality) to 7 (highest possible score) and are ranked as ‘very good/excellent’ score ≥5; ‘good’ score 3·5 ≤ and <5; ‘acceptable’ score 2·5 ≤ and <3·5; and ‘poor’ score <2·5^(^^5^^)^. In order to estimate a mean correlation per micronutrient for the included studies, the correlation coefficient from each study was initially multiplied by its quality score. Next, the sum of the weighted correlations was divided by the sum of the quality scores to provide a correlation coefficient that was adjusted for the study's methodological quality. Mean weighted correlation coefficients were only calculated for micronutrients with correlations available from three or more studies^(^^34^^)^. This allows for concurrent analysis of multiple validation studies and gives an estimate of a mean correlation coefficient per micronutrient for a given dietary assessment method^(^^5^^)^. The intake method was rated as poor when the correlation was <0·30, acceptable between 0·30 and 0·50, good between 0·51 and 0·70, and correlations >0·70 were very good^(^^5^^)^.

## Results

### Study selection

**Fig. 2.**
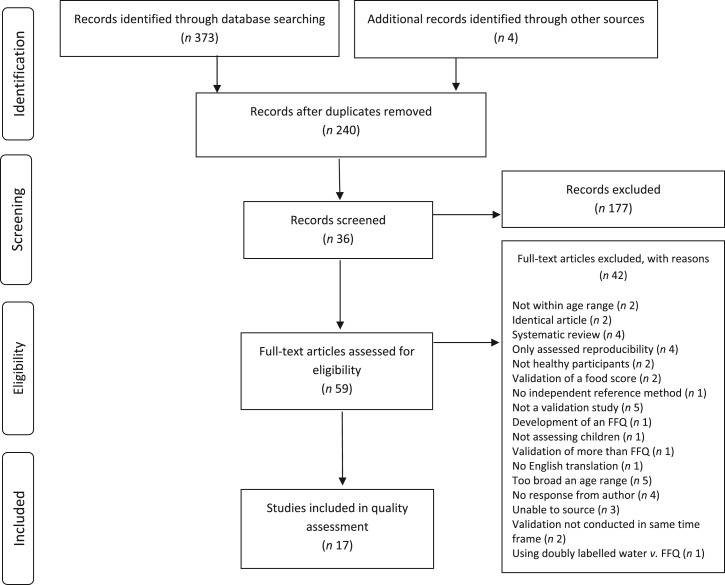
Selection process flow of articles identified that assess validity of FFQ methods in children aged 12–36 months.

A total of 373 articles were identified (Fig. 2). Following removal of duplicates, 236 articles unique by title and abstract remained for review. Application of inclusion and exclusion criteria resulted in fifty-nine articles being selected for full-text review. Thirty-nine studies were included for quality appraisal. All studies were cross-sectional in their design, and thus classified as level IV evidence^(^^35^^)^. Following quality appraisal twenty-two studies were excluded, leaving seventeen articles^(^^11^^,^^15^^–^^24^^,^^26^^,^^27^^–^^29^^,^^31^^,^^37^^)^ identified as assessing the validity of an FFQ against a dietary reference instrument in children 12 to 36 months of age.

Nine of the publications reported results from North American countries (USA and Canada)^(^^16^^–^^20^^,^^23^^,^^24^^,^^28^^,^^29^^)^, five from the UK and Europe^(^^11^^,^^15^^,^^21^^,^^27^^,^^30^^)^, and three from New Zealand^(^^26^^,^^31^^,^^38^^)^. The number of participants ranged from seventeen^(^^16^^)^ to 240^(^^19^^)^, with two studies presenting data from large cohorts: The Iowa Fluoride Study^(^^23^^)^ and The IDEFICS Study (Identification and prevention of Dietary- and lifestyle-induced health EFfects In Children and infantS)^(^^37^^)^.

### Characteristics of included studies

Characteristics of each of the seventeen included validation studies are described in Table 1. Fourteen studies considered the validity of the FFQ to assess nutrient intakes^(^^11^^,^^15^^–^^24^^,^^27^^,^^28^^)^, and three studies considered values on the validity of the FFQ to assess food or food group(s)^(^^29^^,^^31^^,^^37^^)^. Two studies assessing nutrient intakes also used biomarkers as an additional reference method^(^^18^^,^^20^^)^. Eleven of the included FFQ were semi-quantitative^(^^11^^,^^15^^–^^19^^,^^21^^,^^23^^,^^27^^–^^29^^)^, five were quantitative^(^^19^^,^^20^^,^^22^^,^^26^^,^^31^^)^, and one recorded frequency of consumption and not portion sizes^(^^37^^)^. The number of food items ranged from seventy-eight^(^^21^^)^ to 191^(^^20^^,^^29^^)^ with an average of 113 food items. Those studies that assessed food and/or food group intakes had between seven^(^^19^^)^ and seventy-seven^(^^22^^)^ food groups. Food intake intervals ranged from intake over the previous 7 d^(^^19^^,^^23^^)^ to over the last year^(^^18^^,^^28^^,^^28^^)^, with the majority describing intake over the last month^(^^17^^,^^21^^,^^24^^,^^27^^,^^29^^,^^37^^)^.

Two studies were grouped according to a reference method that reflected long-term intake (7-d WFR)^(^^11^^,^^15^^)^. Ten studies were grouped according to a reference method that reflected short-term intake where four applied 24-HR^(^^17^^,^^18^^,^^24^^,^^27^^)^ and five applied WFR^(^^19^^–^^23^^)^, one of these being online^(^^22^^)^. One study utilised biomarkers as a reference method^(^^28^^)^. Among the seven studies that used WFR, the number of recorded days varied from 3 to 7 d^(^^11^^,^^15^^,^^19^^,^^20^^–^^22^^,^^29^^)^. The number of repeated 24-HR ranged from 2^(^^24^^,^^37^^)^ or 3^(^^17^^,^^20^^,^^27^^)^ days of non-consecutive administration. Eleven studies were self-administered^(^^11^^,^^15^^,^^17^^–^^19^^,^^22^^,^^23^^,^^27^^–^^29^^,^^37^^)^, by a parent or equivalent proxy reporter, and six studies were interviewer administered^(^^16^^,^^20^^,^^21^^,^^24^^,^^26^^,^^31^^)^. Methods of portion size estimation ranged from household measures/standard portion sizes^(^^11^^,^^15^^,^^21^^,^^24^^,^^27^^,^^28^^)^ to portion sizes derived from national nutrition survey data^(^^17^^,^^22^^,^^29^^)^. Three studies did not describe portion estimation^(^^18^^,^^20^^,^^23^^)^, and two studies used a unique ‘palm’ measurement^(^^26^^,^^31^^)^. Of the thirteen studies that calculated food intakes into nutrient intakes, six reported using national food composition databases (e.g. United States Department of Agriculture)^(^^11^^,^^15^^,^^22^^–^^24^^,^^28^^)^, and two used other food composition databases (e.g. Harvard Nutrient Database)^(^^17^^,^^18^^)^. Although not the primary aim of the validation study, two studies^(^^16^^,^^18^^)^ examined whether there were any differences between sex and care status (i.e. in child care or at home) when comparing mean nutrient intake values.

### Statistical analysis

Statistical analyses used in the assessment of FFQ validity, and in some cases reproducibility, are described in Table 1. All included studies calculated differences in means and/or mean comparisons. Pearson or Spearman's correlation coefficients were calculated in all studies. Paired Student's *t* tests were used evaluate whether there was any difference between the mean nutrient and food intakes determined by the two assessment methods^(^^18^^)^. Factors that affect the validity of a dietary assessment instrument included: population characteristics, acceptability of the reference method data, FFQ design/quantification, quality control and data management^(^^5^^,^^33^^)^.

The calculation of weighted correlation coefficients allowed comparison with the other included studies. Here, correlation coefficients between 0·51 and 0·7 are considered good^(^^5^^,^^7^^)^. Four studies considered crude correlation coefficients^(^^16^^,^^19^^,^^22^^,^^23^^)^, whilst seven studies adjusted nutrients using energy-adjusted values^(^^11^^,^^15^^,^^17^^,^^18^^,^^20^^,^^21^^,^^28^^)^, and three studies calculated de-attenuated values to account for measurement error^(^^26^^,^^27^^,^^31^^)^ or intra-class correlations^(^^24^^)^. All six studies that performed cross-classification analysis ranked participants by using the same or adjacent quartile. Three of these studies^(^^11^^,^^15^^,^^26^^)^ assessed the classification of participants according to their nutrient intakes and three studies^(^^29^^,^^31^^,^^37^^)^ assessed the classification of participants according to their food or food group intakes. Weighted κ was calculated in two studies that considered food intakes^(^^19^^,^^23^^)^. Here, four categories were used to calculate κ statistics and classify food intake data.

Two studies^(^^24^^,^^26^^)^ assessed the reproducibility of the FFQ for estimating dietary intake patterns and estimation of reproducibility of nutrient intakes was achieved by calculating correlation coefficients and intra-class correlations. Acceptable intra-class correlations ranged from >0·4^(^^7^^,^^26^^,^^39^^)^ to 0·7^(^^24^^)^ when establishing test–retest reliability of the FFQ. In order to test reproducibility, five^(^^17^^,^^24^^,^^26^^,^^29^^,^^31^^)^ studies administered the FFQ on two occasions. Intervals between test and retest ranged from 2 weeks^(^^29^^)^ to 1 month^(^^17^^,^^24^^,^^26^^,^^31^^)^. One study^(^^17^^)^ administered the FFQ on two occasions, 1 month apart but did not report on the statistical analysis used for reproducibility.

### Results of individual studies by validation method used

Included reviews were analysed according to the reference method used (i.e. WFR, 24-HR or biomarker) and whether the tool reflected long-term or short-term intake.

#### FFQ *v.* 24-h recalls

Five studies^(^^17^^,^^18^^,^^24^^,^^27^^,^^37^^)^ used 24-HR as their reference method to validate an FFQ. In all studies the FFQ overestimated median/mean nutrient intake estimates but could provide reliable estimates of nutrient intakes in young children with good agreement when compared with the 24-HR (Table 1). Nutrient correlations that were energy-adjusted or de-attenuated (to reduce dependency on between-person variation) were found to have higher correlation coefficients compared with crude values. Cross-classification into low, medium and high consumers was moderate (>30 % classification into the same quartile). One study^(^^24^^)^ assessed repeatability/reproducibility using a 24-HR as a reference tool. Correlations for most nutrients were >0·70, indicating low within-person variation.

#### FFQ *v.* food record (±weighing)

Eleven studies used WFR as their reference method to validate an FFQ^(^^11^^,^^15^^,^^16^^,^^19^^,^^20^^,^^21^^,^^22^^,^^23^^,^^26^^,^^29^^,^^31^^)^. Ten studies that estimated nutrient intakes found that the FFQ tended to overestimate intakes (Table 1) but found good correlations (>0·4)^(^^7^^)^ between the FFQ and WFR for most nutrients, energy intakes and food intakes. The included FFQ mostly indicated a moderate ability to rank infants according to their nutrient intakes, with two studies by Andersen *et al*.^(^^11^^,^^15^^)^ showing that the ability of the questionnaire to rank infants according to their intakes increased when using nutrient density values over absolute values.

#### FFQ *v*. biomarker

Using biomarkers as the reference method was less frequent. Three studies used biomarkers^(^^15^^,^^24^^,^^35^^)^. Two articles^(^^18^^,^^20^^)^ presented validation of an FFQ using biomarkers and a second dietary assessment instrument (24-HR or WFR) as reference methods. The biomarkers analysed included: total lipids, plasma levels of vitamins C, D and E, retinol and β-carotene^(^^18^^)^, serum markers of Fe^(^^20^^)^ and fatty acid composition measured in erythrocytes^(^^28^^)^.

#### Evaluation of food or food groups

Using a semi-quantitative FFQ excellent reliability and adequate validity were seen in assessing food choices of low-income children^(^^29^^)^, with low levels of agreement and limited ability to rank children according to intakes of food groups^(^^37^^)^. More recently, in Otago, New Zealand, a semi-quantitative FFQ displayed good validity (*r* 0·52) and high reproducibility in the identification of dietary patterns, and in ranking the diets of toddlers when compared with a 5-d WFR. The FFQ overestimated energy and nutrient intakes and cannot measure absolute intakes, but could be used to identify toddlers at extreme ends of intake distribution^(^^26^^,^^31^^)^.

### Additional analysis: quality assessment

A summary of the quality assessment of the seventeen included studies are shown in Table 2. Using the reduced summary score^(^^32^^)^, one validation study that assessed nutrient intakes received a low quality ranking^(^^19^^)^ and one study that assessed food intake received a low quality ranking^(^^37^^)^. The remaining fifteen studies received high quality rankings. Criteria that reduced the quality of the study included the number of food items in the FFQ (<70 food items is likely to reduce the quality of the nutrition information), and if the FFQ was self-administered.

**Table 2.** Quality scores using methods described by Dennis et al.^(^^32^^)^ and the EURopean Micronutrient RECommendations Aligned (EURRECA) scoring tool^(^^5^^)^

Study	Quality level (Dennis *et al.*)^(^^32^^)^*	Quality score^(^^5^^)^†	EURRECA classification
Nutrient intake			
Williams & Innis^(^^20^^)^	High	6·0	Excellent
Watson *et al.*^(^^26^^)^	High	5·0	
Sochacka-Tatara & Pac^(^^27^^)^	High	5·0	
D'Ambrosio *et al.*^(^^24^^)^	High	4·5	Good
Marriott *et al.*^(^^21^^)^	High	4·0	
Andersen *et al.*^(^^11^^)^	High	3·5	
Andersen *et al.*^(^^15^^)^	High	3·5	
Parrish *et al.*^(^^18^^)^	High	3·5	
Orton *et al.*^(^^28^^)^	High	3·0	Acceptable
Iannotti *et al.*^(^^16^^)^	High	3·0	
Blum *et al.*^(^^17^^)^	High	3·0	
Marshall *et al.*^(^^19^^)^	Low	3·0	
Vereecken *et al.*^(^^22^^)^	High	3·0	
Rankin *et al.*^(^^23^^)^	High	2·5	Poor
Food or food group intake			
Klohe *et al.*^(^^29^^)^	High	NA	NA
Bel-Serrat *et al.*^(^^37^^)^	Low	NA	NA
Mills *et al.*^(^^31^^)^	High	NA	NA

NA, not available, fewer than three studies found.

* Dennis *et al.*^(^^32^^)^ quality level: high (≥5); low (<5).

† EURRECA quality score: very good/excellent (≥5); good (3·5≥ to <5); acceptable/reasonable (2·5≥ to <3·5); poor (<2·5).

Using the EURRECA scoring system^(^^5^^)^, fourteen studies assessed nutrient intakes, with quality scores ranging from 2·5 to 6·0 (maximum 7·0). The average quality score was 3·8, with a median of 3·5. Table 2 illustrates the classification of the included studies according to their reference method and methodological quality, with three studies^(^^20^^,^^26^^,^^27^^)^ (21 %) rating as very good/excellent, five studies^(^^11^^,^^15^^,^^18^^,^^21^^,^^24^^)^ (36 %) as good quality, five studies^(^^16^^,^^19^^,^^22^^,^^23^^,^^28^^)^ (36 %) having an acceptable quality, and one study^(^^23^^)^ (7 %) having a poor quality rating. ‘Good’ quality scores were seen in the validation studies where FFQ were compared with a reference method that was reflective of long-term intakes, and a majority (58 %) of validation studies where the FFQ was compared with a reference method that was reflective of short-term intakes were either ‘good’ or ‘very good’. Factors affecting the EURRECA quality assessment score^(^^5^^)^ were the statistical analyses used and data collection via interviewer-administration. Calculation of energy-adjusted^(^^11^^,^^15^^,^^17^^,^^18^^,^^21^^,^^24^^,^^28^^)^, de-attentuated (to reduce the dependency on between-person variation)^(^^26^^,^^27^^)^, or intra-class correlation coefficients increased quality scores^(^^24^^)^.

#### Concurrent validation analysis

Table 3 displays concurrent analysis of the included studies where a mean correlation coefficient per nutrient for each dietary assessment method was calculated by multiplying the correlation coefficient by their quality assessment score. This was completed for the EURRECA priority micronutrients and those studies that met the criteria of having nutrient correlations from at least three studies^(^^40^^)^. Micronutrients with a sufficient number of studies to be included (≥3 studies), and where the validation reference method reflected short-term intakes (<7 d), were vitamin B_12_^(^^17^^,^^21^^,^^24^^,^^26^^,^^27^^)^, vitamin C^(^^17^^,^^20^^,^^21^^,^^24^^,^^26^^,^^27^^)^, vitamin D^(^^19^^,^^21^^,^^27^^)^, Ca^(^^17^^,^^19^^–^^22^^,^^24^^,^^26^^,^^27^^)^, Fe^(^^17^^,^^20^^,^^21^^,^^24^^,^^26^^,^^27^^)^ and Zn^(^^17^^,^^21^^,^^26^^,^^27^^)^. Fibre ^(^^17^^,^^20^^,^^22^^,^^24^^,^^26^^,^^27^^)^ and vitamin E^(^^17^^,^^18^^,^^21^^,^^27^^)^ also had sufficient studies to allow for concurrent analysis. There were insufficient data available for the analysis of two (20 %) micronutrients: folate and Cu. Using the EURRECA scoring tool classifications, correlations were acceptable for vitamin B_12_ (0·30), vitamin A (0·34) and Ca (0·49) using FFQ *v*. 24-HR whilst Fe showed a poor correlation (0·29) on validation. Acceptable correlations were seen for vitamin C (0·32) and Fe (0·39), and Ca presented a good correlation (0·51) using FFQ *v.* WFR. The intake method was rated as ‘good’ when the mean correlation coefficient weighted by the quality criteria score was at least 0·5. The number of studies that used a validation reference method that reflected long-term intakes (>7 d) were insufficient for concurrent analysis (<3 studies per micronutrient).

**Table 3.** Classification of dietary assessment methods for infants aged 12–36 months according to the weighted mean of the correlations of micronutrients with three or more studies available (separate comparisons of those studies reflecting long-term and short-term intakes or comparison of FFQ with a reference method)

	Correlation*				
Micronutrient	Long-term intake (>7 d)	Short-term intake (<7 d)	FFQ *v*. WFR	FFQ *v*. 24HR	FFQ *v*. BM
Vitamin B_12_	NA	P – 0·20 (5 studies)	NA	A – 0·30 (3 studies)	NA
Folate	NA	NA	NA	NA	NA
Vitamin C	NA	A – 0·31 (7 studies)	A – 0·32 (3 studies)	A – 0·34 (4 studies)	NA
Vitamin D	NA	P – 0·13 (3 studies)	NA	NA	NA
Ca	NA	G – 0·51 (8 studies)	G – 0·51 (7 studies)	A – 0·49 (3 studies)	NA
Fe	NA	A – 0·33 (6 studies)	A – 0·39 (5 studies)	P – 0·29 (3 studies)	NA
Zn	NA	P – 0·15 (4 studies)	NA	NA	NA
Cu	NA	NA	NA	NA	NA
Fibre	NA	P – 0·29 (6 studies)	A – 0·31 (3 studies)	P – 0·24 (3 studies)	NA
Vitamin E	NA	P – 0·11 (4 studies)	NA	P – 0·23 (3 studies)	NA

WFR, weighed food record; 24HR, 24 h recall; BM, biomarker; NA, not available, fewer than three studies found.

* Correlation: G, good (0·51–0·70); A, acceptable (0·30–0·50); P, poor (<0·30).

## Discussion

In this review, using standardised quality assessment methods, we evaluated seventeen studies reporting on the validity of FFQ as a method for assessing food and nutrient intakes or dietary patterns in 12- to 36-month-old children. From the identified studies^(^^11^^,^^15^^–^^20^^,^^21^^,^^23^^,^^24^^,^^26^^,^^27^^–^^31^^,^^38^^)^, semi-quantitative FFQ were shown to be valid and reproducible instruments in children as young as 1 year of age, generating adequate estimates specifically for Ca, vitamin C and Fe, with results similar to those seen in older children and adolescents^(^^18^^,^^22^^)^.

FFQ are used to assess dietary intake due to their practicality, relative ease of administration, low participant burden, ability to assess intake over a prolonged period of time, and lower associated costs^(^^41^^,^^42^^)^. However, there are limited FFQ that have been specifically validated in 12- to 36-month-old children. In the present review, the methodological qualities of FFQ were considered in conjunction with analysis of weighted correlation coefficients where higher weights were given to studies that employed higher quality methodologies^(^^5^^,^^34^^)^. Qualities included data collection methods, administration, seasonality, sample size, supplement use and statistics.

It is estimated that at approximately 7 to 8 years of age children become aware of their own food intake. Prior to this age the cognition and attention span required to perceive time frames, have knowledge of foods, recall food intake, and self-report are not sufficiently developed^(^^1^^)^. Other explicit issues that arise in this age group of interest relate to the change in dietary practices seen across the age range and the variability in information provided by parent or proxy reporter, on foods that are eaten outside of their supervision, especially when the child is in day care.

The ability of FFQ to rank nutrient and energy intakes is improved through providing detailed quality information which can be achieved through interviewer administration^(^^21^^)^.The majority (71 %) of the included FFQ were self-administered by a parent or proxy reporter, similar to that seen in reviews conducted in wider age groups^(^^34^^,^^43^^)^. Cade *et al*.^(^^7^^)^ reported an increase in correlation coefficients when the FFQ was interviewer-administered, with the exception of vitamin C, in comparison with those that were self-administered. This is especially relevant in the age group in question, where all information is obtained from a parent or proxy-reporter. There is a need for further studies designed to evaluate the accuracy of parental-reported intakes in larger, ethnically diverse populations, using different dietary assessment methods^(^^44^^)^.

Estimation of portion size appears to have some advantage over using average or specified portion sizes, with higher measures of agreement between FFQ and reference method (*r* 0·5–0·6) and higher correlation coefficients when assessing repeatability^(^^33^^)^. FFQ are seen to commonly overestimate energy intake, which is especially apparent in this population of interest^(^^11^^,^^15^^,^^17^^,^^18^^,^^21^^,^^24^^)^. This could be attributed to the fact that parents/caregivers may not adequately take into account the small portion sizes consumed by their children and that young children often ‘taste’ many foods without consuming full portions, leading to the inclusion of too large a portion size for some foods^(^^11^^,^^18^^)^. Many of the included studies assessed wider age ranges, i.e. beyond 12 to 36 months, which, as identified in a recent validation study performed in New Zealand, may act to improve validity of the FFQ as older children are more likely to eat meals that are similar to that of the family member or adult completing the FFQ^(^^26^^)^. Improvements in validity and bias could be seen through reducing the number of food items in the FFQ, shortening the reporting period, or adjusting portion sizes to more closely reflect those consumed by a young child^(^^44^^)^. This unique method has been explored in a study performed in 12- to 24-month-old New Zealand children where the amount of food offered and the amount eaten were recorded separately to encourage parents to differentiate between the two, and portion sizes were described according to the child's ‘palm volume’. This FFQ showed acceptable to good validity and high reproducibility in the assessment of dietary patterns and ranking nutrient intakes^(^^26^^,^^31^^)^.

In a systematic review by Henríquez-Sánchez *et al*.^(^^43^^)^, an improvement in correlation coefficients (*r* 0·52) was seen when the number of food items included in the FFQ was greater than 100 (*r* 0·47). The average number of food items used in the present review was 113. Estimation of supplement use should be considered when evaluating nutrient intake. Information on supplements should be included in dietary assessment with emphasis on the type and dose used. Data from FFQ and reference methods correlated better when supplement intake was captured^(^^43^^)^. Supplement use was acknowledged in one study^(^^20^^)^ and seasonality in another^(^^24^^)^, but were not considered in the statistical analysis.

All studies calculated Pearson or Spearman's correlation coefficients (Table 1). Calculation of correlation coefficients does not measure agreement between the two methods of dietary assessment, only the degree in which the two methods are related^(^^45^^)^. Their usefulness increases if used in conjunction with an alternative method such as Bland–Altman which provides an analysis of how well the FFQ and reference method agree on average^(^^45^^)^. Other methods such as limits of agreement can be used to provide information on reliability and the direction and consistency of bias and the magnitude of errors between the two assessment methods^(^^7^^,^^33^^)^. It is difficult to summarise the correlation coefficients, agreement of validity and reproducibility of the included FFQ; therefore the present review should be used as a description of included FFQ, with potential for further meta-analyses.

Using 24-HR as the dietary reference method, FFQ were found to be a suitable tool for ranking children according to nutrient intakes (*r* 0·46), with stronger correlations in foods consumed more frequently^(^^27^^,^^37^^)^. This highlights the difficulties with episodically consumed food items, as seen in the high day-to-day variability of a young child's diet^(^^18^^,^^37^^)^. Unadjusted FFQ nutrient estimates were larger than unadjusted nutrient estimates from multiple 24-HR and additional analysis of children that regularly received meals and snacks from other caregivers alongside parents revealed no apparent compromise or differences in correlations^(^^18^^)^.

Using WFR as the reference method to assess long-term intakes, correlations were found to increase using nutrient density values over absolute intakes, but the FFQ had a low to moderate ability to rank children according to intakes of nutrients and foods^(^^11^^)^. WFR are not affected by the same errors, such as portion size estimation, and memory lapses, as the FFQ^(^^39^^)^. The FFQ was found to be a useful tool for estimating short-term energy and nutrient intakes in healthy infants (at a group level)^(^^21^^,^^22^^)^. Marriott *et al.*^(^^21^^)^ found that differences in micronutrient intakes were partly explained by changes in the consumption of milk between the two dietary assessments and by the different nutrient compositions of cows’ milk and formula^(^^21^^)^. This underestimation of Ca intake by the FFQ has been reported in three studies within this age group^(^^19^^,^^21^^,^^46^^)^.

The use of FFQ to provide estimates of beverage intake has not been widely investigated. Marshall and Rankin concluded that a quantitative FFQ could be used to provide relative estimates of beverage, Ca, vitamin D and fluoride intakes in this age group^(^^19^^,^^23^^)^ and higher correlations were seen at younger ages when the diet was more limited (*r* 0·85 at 6 months *v*. *r* 0·65 at 60 months)^(^^23^^)^.

The present review included correlations from three studies using a biomarker for validation^(^^18^^,^^20^^,^^28^^)^. In the assessment of specific nutritional status, Williams & Innis^(^^20^^)^ showed that a semi-quantitative FFQ could be a useful tool in assessing Fe status in infants at a group level (energy adjusted *r* 0·71), but could result in underestimation of infants deemed to be at high risk of poor Fe status^(^^18^^,^^20^^)^.

### Evaluating quality assessment

Where correlations for a given nutrient were available from three or more studies, quality-adjusted correlations were calculated. Higher weighted mean correlations were seen in studies that used WFR as the reference method for Ca, Fe and fibre when compared with other methods. This may be a reflection of the fact that a greater number of studies (60 %) used WFR as a reference method. The highest correlation coefficient weighted by quality was 0·51. There were not sufficient data to conduct the analysis for the remaining micronutrients, and only six out of the ten EURRECA priority nutrients could be assessed. This continues to remain a concern in this age group, where valid nutrient intake estimates could not be calculated. FFQ validation studies that assessed long-term intakes or used biomarkers as the reference tool were based on one or two studies, making them insufficient to reach any conclusion (Table 3).

### Limitations

There was a lack of data available to assess the ability of the FFQ in providing adequate estimates for several of the micronutrients highlighted in the present review (Table 3). The heterogeneity in the study designs, methods, outcomes and assessment tools made comparisons difficult, therefore the data were narratively synthesised and described. Due to natural variation, biomarkers may not always be a suitable option for comparison^(^^18^^)^ and few studies validating FFQ using biomarkers were available for inclusion in the present review which would act to reduce correlated errors associated when the reference method is based on self-reporting^(^^47^^)^. Studies that assessed the validity of energy intake measurements using doubly labelled water did not meet our inclusion criteria. Due to the specific range of interest, several studies that reported over a wider age range were excluded as reviewers were unable to extract these data. Correlation coefficients of the included studies were used for analysis and quality assessment in the present review; this limits the interpretation of the review as correlation coefficients only measure the degree to which the two assessment methods are related in a validation study, and not the agreement between the methods^(^^48^^)^. De-attenuation and energy adjustment have strong implications for correlation coefficients and make it difficult to compare and draw conclusions. Only validation studies written in English were included for analysis. This may have led to the exclusion of reliable validation studies from other countries.

### Conclusion

This systematic literature review presents a summary of the quality of FFQ validation studies in children aged 12 to 36 months. The included studies and quality assessment have provided information on aspects of FFQ design that increase validity, such as the number of items included, portion size estimations, appropriate food choices, administration method, validation and reproducibility methods, pre-testing, supplement use, seasonality and the statistical analyses. Semi-quantitative FFQ were shown to be valid and reproducible when estimating dietary intakes at a group level, and are an acceptable instrument for estimating intakes of Ca, vitamin C and Fe in children 12 to 36 months of age. There is insufficient evidence for the evaluation of the validity of micronutrients such as folate, vitamin D, Zn and Cu in this population. Using the results of the included studies; meticulously designed and validated FFQ may be acceptable in estimating intakes of a number of important micronutrients in this age group.

Children aged 12 to 36 months would benefit from further validation studies using appropriate population-specific tools addressing areas highlighted in this review that are unique to dietary assessment in young children. Such areas include further development on portion size estimation, capturing irregular eating patterns, overcoming administration errors with the implementation of computer-assisted methods or the development of novel tools to provide evidence for further validation studies of appropriate population-specific tools, alongside the identification, management and primary prevention of diet-related disease processes.
